# The Role of Hemp (*Cannabis sativa* L.) as a Functional Food in Vegetarian Nutrition

**DOI:** 10.3390/foods12183505

**Published:** 2023-09-20

**Authors:** Gianluca Rizzo, Maximilian Andreas Storz, Gioacchino Calapai

**Affiliations:** 1 Independent Researcher, Via Venezuela 66, 98121 Messina, Italy; 2 Department of Internal Medicine II, Centre for Complementary Medicine, Medical Center—University of Freiburg, Faculty of Medicine, University of Freiburg, 79106 Freiburg, Germany; maximilian.storz@uniklinik-freiburg.de; 3 Department of Chemical, Biological, Pharmaceutical and Environmental Sciences, University of Messina, 98166 Messina, Italy; gcalapai@unime.it

**Keywords:** *Cannabis sativa* (industrial hemp), hemp seed oil, hemp seed proteins, hemp seed minerals, hemp seed dietary supplementation, hemp seed-based food, bioactive peptides, antioxidant activity, vegetarian diet, plant-based diet

## Abstract

Recently, there has been a renewed interest in *Cannabis sativa* and its uses. The recreational use of inflorescences as a source of THC has led to the legal restriction of *C. sativa* cultivation to limit the detrimental effects of psychotropic substance abuse on health. However, this has also limited the cultivation of textile/industrial varieties with a low content of THC used for textile and nutritional purposes. While previously the bans had significantly penalized the cultivation of *C. sativa*, today many countries discriminate between recreational use (marijuana) and industrial and food use (hemp). The stalks of industrial hemp (low in psychotropic substances) have been used extensively for textile purposes while the seeds are nutritionally versatile. From hemp seeds, it is possible to obtain flours applicable in the bakery sector, oils rich in essential fatty acids, proteins with a high biological value and derivatives for fortification, supplementation and nutraceutical purposes. Hemp seed properties seem relevant for vegetarian diets, due to their high nutritional value and underestimated employment in the food sector. Hemp seed and their derivatives are a valuable source of protein, essential fatty acids and minerals that could provide additional benefit to vegetarian nutrition. This document aims to explore the information available in the literature about hemp seeds from a nutritional point of view, highlighting possible beneficial effects for humans with particular attention to vegetarian nutrition as a supplemental option for a well-planned diet.

## 1. Introduction

Hemp (*Cannabis sativa* L.) is an annual herbaceous plant from the Cannabaceae family [1]. Its use by humans is widely documented for textile and food purposes [2]. Although archaeobotanical findings show that *C. sativa* was indigenous to Europe [3], the first evidence of its cultivation and domestication suggests that it was introduced to Europe from Asia more than 4000 years ago during the Bronze Age [4]. 

Hemp is one of the oldest crops that has been domesticated by humans [5]. While the stems have been widely used for the production of ropes and fabrics, the seeds have been used as a food, thanks to their remarkable nutritional properties. However, this intended use seems to be more relevant in recent times, while in ancient times hemp was mainly used for textile purposes and only marginally in medicine and as traditional food [6,7]. 

However, the narcotic or recreational use of the inflorescences, which contain psychoactive cannabinoids, led to the ban on *C. sativa* in the early 1930s from many countries. For a long time, its use for any purpose was prohibited starting from USA through the Marijuana Tax Act. 

In fact, the ban did not distinguish crops by intended use (textile/food or recreational use) [8]. Its classification has been frequently based on the content of psychoactive substances, distinguishing the *C. sativa var. Indica* (marijuana) and *C. sativa var. sativa* (hemp) [9]. Nowadays, Marijuana is used as a substance of abuse and for pharmacological purposes, while hemp is used for food, textile and industrial purposes. These varieties are interfertile and therefore belong to the same botanical species [10]. Although in the USA the ban was temporarily lifted during the Second World War, many of the industrial uses of hemp had already been replaced by other options such as oils of different origins, cotton and synthetic fiber for textile purposes.

Currently, many countries such as Canada, USA, Australia, China, and most of the European countries (Austria, France, Spain, Great Britain, etc.) allow the cultivation of low-THC cultivar (<0.3% *w*/*w*), with a renewed interest in the last 20 years towards hemp for nutritional applications [6,11]. In some countries, there are more stringent limits, such as in Italy where the permitted limit of THC content is set to 0.2% [12]. Canada, in particular, was the first Western country to reintroduce the cultivation of hemp, followed only later by the USA and some European countries. In Europe, the major hemp producers are France, Spain, Great Britain and Austria [11]. China covers 50% of the world’s production of hemp fiber [13].

**Figure 1:**
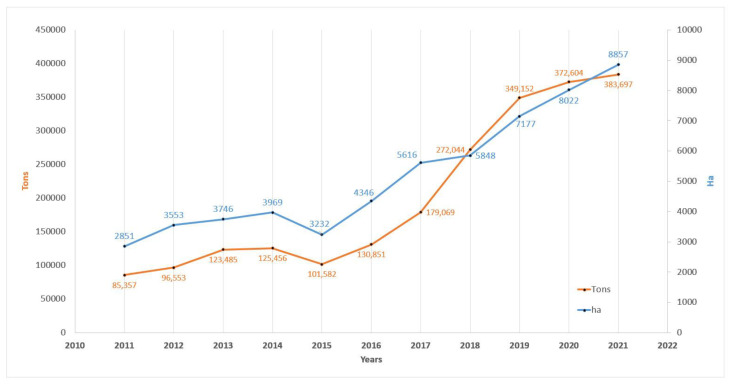
Area harvested (ha) and production (tons) of hemp seeds in Europe from 2011 to 2021.

As food, the use of hemp takes place through the use of the seeds as a whole, reduced to flour or transformed, as for the lipid components, following the extraction of the oil. These three forms of use have shown promise, and the recent revival of interest in hemp for nutritional purposes has suggested that some properties of hemp seeds can be integrated into human nutrition. In particular, hemp could be of interest in the case of vegetarian nutrition thanks to the high content of proteins and essential fatty acids. Recently, in Europe, especially in Germany and the Netherlands, the consumption of hemp seeds for nutritional purposes has almost doubled subsequent to the increased marketing of hemp-based products [14]. Data from the Food and Agriculture Organization Corporate Statistical Database suggest that the cultivation of hemp for obtaining seeds in the last 10 years (2011–2021) in Europe has increased from 2851 ha to 8857 ha, with its production increasing from 85,357 to 383,697 tons per year (Figure 1) [15].

The interest in plant-based diets is growing thanks to their more favorable environmental and ethical impact and multiple beneficial effects on health [16]. Vegetarian diets, which include strict vegetarian or vegan diets and lacto-ovo vegetarian diets, can be sustainable for health if well planned [17]. Although many nutrient-rich plant sources are readily available as food, hemp could provide additional nutritional benefits and increase the number of options available in diets that exclude animal sources. Hemp seeds are a nutritional source rich in high-value nutrients such as proteins, with good digestibility and essential amino acids, polyunsaturated fatty acids, fiber, minerals and vitamins. Most of these molecules are valuable nutrients that support a well-planned vegetarian diet but have a broad application for a healthy diet in the general population. Furthermore, hemp seeds are also a rich source of health-promoting antioxidants and phytochemicals that may be useful in supporting health and aiding the transition to plant-based diets. With the growing interest in hemp for food purposes, industry interest is also growing, highlighting promising uses in the bakery, supplement and functional food industries.

The purpose of this review is to describe the current knowledge about the nutritional properties of hemp, with a particular focus on its role in vegetarian diets. Table 1 resumes hemp’s features that can be of interest in a vegetarian diet.

## 2. Harmful Effects of *C. sativa* on Health

The use of cannabis for recreational purposes causes cognitive changes, anxiety, psychotic symptoms, panic, and numerous other psychotropic effects such as relaxation and euphoria that stimulate its consumption [18]. Its use can create addiction and increase the risks associated with it [19]. These effects are mediated by THC, whose concentrations are related to motor alterations, and reductions in attention and reaction speed, which can lead to dangerous consequences for health [20]. The consequences of cannabis consumption are significantly correlated to the risk of road accidents, with a percentage of between 4 and 14% of drivers testing positive following traffic crashes [21,22]. Considering that the illicit use of cannabis occurs mainly through smoking, marijuana users show more evident pathological signs of airway alterations than tobacco smokers [23].

Regarding the reproductive system, THC diffuses easily across the placenta and mammary gland, and recreational cannabis use appears to be related to a higher incidence of preterm birth [24].

Cannabis smoking may also be linked to the incidence of cardiovascular disease in adults [25].

However, the psychotropic effects of cannabis are linked to the consumption of cultivars with a high THC content, whose cultivation and use are illegal. The varieties permitted for cultivation for food purposes have negligible concentrations of psychoactive substances, which do not raise the aforementioned concerns. Furthermore, THC production occurs in the inflorescences and is not found in hemp seeds, which are the anatomical part of the plant used as food.

The regular and consistent dietary consumption of hemp does not raise concerns about THC levels and related psychotropic problems or other adverse health effects [26].

## 3. Nutritional Aspects

As regards their nutritional use, hemp seeds, and, therefore, the fruits of *C. sativa*, are the part of the plant used most for human consumption. From a botanical point of view, hemp seed is an achene, i.e., a dried fruit whose hardened pericarp does not adhere to the internal seed, as for quinoa, amaranth, buckwheat and strawberry achenes [27]. In this paper, we refer to hemp seeds when we generally speak of hemp as a plant material for nutritional purposes, unless otherwise specified. Although other anatomical parts of the plant such as flowers and leaves are potentially edible, the use of hemp for nutritional purposes which does not concern the seed is negligible and this prevents the derived food products from containing traces of cannabinoids and, therefore, being prohibited by specific legislation [28]. The great interest in hemp for nutritional purposes emerged rather recently, since in the past centuries it had been used mostly for textile purposes and the seeds were considered a waste [29]. 

There are nearly 50 hemp cultivars grown for nutritional interest with a varying macronutrient composition. The protein content does not exceed 30%, with the dietary fiber content ranging between 30 and 40% and a lipid content of approximately 25–30% [30]. Dietary fibers are concentrated in the external integuments of the seed, and hulling allows the elimination of most of them [31]. Hulled seeds show fat and protein contents over 46% and 35%, respectively [11]. On the other hand, the amount of available carbohydrates is usually very low and negligible since its polysaccharide component is almost exclusively composed of dietary fiber (refer to the specific section below). However, the interesting nutritional aspects of hemp also include the presence of bioactive peptides with an antioxidant effect, and other phytochemicals such as polyphenols and sterols [32]. Hemp seeds are very versatile and promising and the nutritional profile of hemp-based products can meet the market’s needs [33]. They can be consumed as a whole, hulled or in the form of oil, flour, or isolated proteins. Seed germination contributes to further modifying the nutritional profile by increasing the bioavailability of the phytochemicals present [34]. This could increase the beneficial effects of the seeds due to the increased anti-inflammatory and antioxidant power. The USDA database is currently the widest archive about food composition [35]. Table 2 shows the composition of hemp seed compared with legumes and other nuts and seeds.

### 3.1. Protein

Generally, hemp seeds are processed as a first step for the extraction of the oil, thus obtaining the defatted meal or seed cake, whose macronutrient composition shows a high protein content. Hemp seeds are a promising source of protein that deserves more attention, as found in recent proteomic characterizations [36]. In an evaluation of the nutritional characteristics of hemp seeds of the Futura 75 cultivar grown in Caserta (South Italy), it was highlighted that the seed flour or the oil meal (the byproduct obtained through the mechanical cold-pressing extraction of the oil), contains more than 30% of protein [37].

The hemp protein is composed of three main fractions: globulins, albumins and minority peptide chains rich in sulfur [38]. Among its notable characteristics, hemp protein exhibits high levels of glutamic acid and arginine, which have sparked interest in this protein source. Arginine accounts for 12% of hemp protein, a markedly higher fraction than other high-protein animal-derived or plant sources such as wheat, soy, egg white and whey, showing up to 7% content [8]. While the effect of arginine on blood pressure via the nitric oxide regulatory pathway is well known [39], it may have a positive effect on athletic performance [40]. Furthermore, hemp has a higher sulfur amino acid content than soy and casein [31]. A high ratio of Arg/Lys in the globulin fraction compared to albumin (4.37 vs. 1.74) may suggest its use for various health purposes [41]. This ratio is also higher than those found in soy or casein [11]. Among the globulins, edestin is a legumin that represents up to 75% of the protein fraction of hemp, followed by a 37% fraction represented by albumin [36]. A third protein fraction is characterized by the presence of a vicilin-like protein: a beta-conglycinin protein that represents about 5% [42]. The highest protein concentration was found in the cotyledons, and to a lesser extent in the hull fraction [42]. The removal of both the hull and oil leads to an increase in the relative protein content to 50% and over [31].

Hemp proteins may exert beneficial effects on human health. A clinical trial is currently underway to evaluate the effects on blood pressure of various protein sources, including hemp protein, and also hydrolysates and derived biopeptides [43]. The results are expected to be available in 2024. Some of these mechanisms will be discussed later.

In a well-planned vegetarian diet, the protein requirement is achieved without great concern [17,44,45]. However, this requires a wide choice of vegetable protein sources every day to favor protein intake in general and all the essential amino acids in particular [46]. Hemp and derived products could help one to obtain an additional protein source with a high biological value. Hemp has been shown to have an amino acid score comparable to egg white protein [8]. The digestibility of hemp proteins increases if the extraction takes place from hulled seeds, with higher values compared to soy isolates [47]. A digestibility value of 97% of hulled hemp seeds was estimated, which was comparable to that of casein [31]. Although dehulling could presumably improve the Protein Digestibility Corrected Amino Acid Score (PDCAAS) value of hemp seeds, the literature is still scarce on this topic, especially regarding products derived from processed hemp seed. It has been shown that the in vitro digestion of isolated hemp proteins showed greater digestibility than isolated soy proteins [47,48].

The protein fraction with the highest biological value is represented by edestin, which has an amino acid composition rich in branched-chain amino acids, methionine, cysteine and aromatic amino acids, which are lowly represented in a vegetarian diet [49,50]. It is found in the aleurone layer of the seed and has a structure similar to soy glycinin [51]. The albumin fraction represents a smaller portion than that of edestin but contains fewer disulphide bonds with a consequently less-compact structure [49]. Furthermore, despite similar emulsifying capacities, hemp albumin shows a greater foaming capacity and solubility than globulin. This minority peptide fraction rich in cysteine does not show inhibiting effects on trypsin, an aspect that increases its digestibility [38,52].

Characteristics of hemp proteins such as solubility, foaming, emulsification, oil-binding, solubility, gelation and film formation are very important factors for the use of hemp in the food-grade industry and were extensively discussed by Wang and Xiong in a recent review [11]. Given the compact structure of hemp proteins, structural modifications through heat, pressure, filtration, enzymatic digestion, acylation, pH shift, irradiation, plasma technology, supercritical carbon dioxide, ultrasound, and electric fields can improve the characteristics that currently limit its industrial use. From a protein point of view, the composition of hemp seeds can be compared to soybean meal, which is often used for the production of plant-based meat alternatives due to its high biological value [31,53].

However, it should be considered that, like many other plant-based nutritional sources, hemp also contains some anti-nutrients that could interfere with the bioavailability of proteins such as phytic acid, tannins and protease inhibitors [54]. In defatted hemp seeds, 4–8 g/100 g of phytic acid, 11–28 U/mg of trypsin inhibitors [55], from 11 to 28 units per milligram of trypsin inhibitors [56] and from 47 to 70 mg per 100 g of saponins [56] may be found. Then again, the concentrations of these substances are comparable to those usually found in legumes and nuts [57]. Its digestibility is comparable to that of legumes but higher than grains [31]. The antinutrient content in hemp seeds is considered relatively low [56]. Furthermore, the removal of the hull allows an increase of almost 10% in the digestibility of hemp protein [31]. Lower amounts of antinutrients have been identified in dioecious varieties compared to monoecious ones [56]. The influence of these substances on human health is much debated and there are also clues to their possible beneficial effect on cancer and metabolic pathologies [58,59,60,61]. The highest concentration of antinutrients appears to be in the external tissues of the seed, and for this reason, digestibility is higher in hulled seeds than whole seeds or from hemp seed cake, with higher PDCAAS values than wheat, lentils and beans but still lower compared to beef [31]. In a comparative study between two cultivars of industrial hemp, USO 21 and Futura 75, following extraction through cold pressing of the seeds, the hemp meal obtained showed a high amount of protein of about 30 g/100 g FW, with a negligible content of antinutrient factors [62]. This finding makes the byproducts of hemp oil a promising source for reuse as food and as a source of protein matter. The limiting amino acid in the case of raw hemp seeds appears to be lysine, as is generally observed for nuts and grains. This is reflected by a rather low lysine score of 0.5–0.62 [31]. Furthermore, it is well known that this amino acid is sensitive to Maillard reactions during cooking [63]. However, hemp can be still consider a high-quality protein source such as casein and soy [64].

#### 3.1.1. Bioactive Peptides

As discussed, hemp is a good source of protein; however, bioactive peptides with various properties, including antioxidant activity, can be obtained from *C. sativa* polypeptides. The most active peptides are those with the lowest molecular weight because they readily interact with the molecular targets, reaching them even more easily by evading intestinal enzymatic hydrolysis. For example, Orio et al. obtained several peptides from the hydrolysis of hemp seed proteins, finding molecules with marked inhibitory properties on angiotensin-converting enzyme (ACE): a useful feature for treating hypertension [65]. Furthermore, peptides obtained from hemp show the inhibition of renin [66] and acetylcholinesterase (AChE) [67].

The AChE-inhibitory action is the same as some drugs usually used in the treatment of Alzheimer’s disease [68]. The hypotensive effects of hemp biopeptides (ACE inhibitor and renin inhibitor) seem to depend on the presence in the peptide chain of prolines and phenylalanine [69]. The AChE-inhibitory action, on the other hand, could also depend on the presence of high-arginine residues, as shown by the hydrolysates tested for this purpose [67]. A clinical trial on the effect of bioactive peptides on hypertension was recently concluded, and its results could clarify the effect of hemp protein and biopeptide consumption on blood pressure [43,70].

The beneficial effects of bioactive peptides obtained from hemp proteins also show other potentially beneficial effects such as antioxidant activity [71], ion scavenger activity through metal-binding [47,72] and the influence on the regulation of cholesterol and glucose concentrations [73,74]. The antidiabetic effect could be mediated by the presence of peptides with an inhibitory effect on alpha-glucosidase, whose interaction involves hydrophobic residues such as leucine and proline [74]. The cholesterol-lowering effect of hemp biopeptides seems to depend on the interaction with the HMGCoAR enzyme which participates in the endogenous biosynthetic pathway of cholesterol [73]. Other statin-like mechanisms need to be elucidated but the interaction with sterol-responsive element binding protein 2, which regulates genes involved in cholesterol homeostasis, could be implicated [67].

#### 3.1.2. Structural Modification of Hemp Protein

Chemical–physical modifications can alter the structural characteristics of hemp protein and improve its rheological properties. Some examples are shown below. 

The ionic bonds of the protein scaffold can be destabilized by treatments that shift from a neutral pH. The molecular unfolding generated by the pH shift can improve the solubility and emulsifying activity of a vegetable protein [75]. 

The acylation and succinylation of amino acid residues are industrial processes widely used to modify the structures of various plant proteins [76]. If used at a pH above 5 they can improve protein solubility. Conversely, extensive modification or a working pH below 5.0 can worsen it. Heat treatment above 30 °C can modulate the molecular bonds of hemp protein extracts, improving their solubility for treatments up to 60 min but with enhancement even for 1–5 min [77]. Heat treatment can be further effective in improving the emulsifying activity of hemp proteins if performed at pH values far from the isoelectric point and, therefore, with both acidic and alkaline pH values [78]. Heat treatment also improves its water-holding capacity [79]. The application of high pressures and pH shifting to hemp seed milk showed an improved oxidative stability of the product [80]. 

Various properties, such as solubility and emulsifying capacity, can be improved by partial hydrolysis with proteolytic enzymes that generate peptides with a reduced molecular weight. However, extensive hydrolysis could induce increased hydrophobicity through peptide aggregation [81]. Again, the working pH plays a key role since enzymatic proteolysis at an alkaline or acidic pH improves emulsion stability, but it worsens at a neutral pH [78].

More modern techniques, such as ultrasonication, appear promising in improving the solubility of isolated hemp proteins, but require further research for researchers to develop optimal conditions of use [11].

### 3.2. Lipids

The oil contained in hemp seeds currently represents the component with the greatest industrial interest. The fat content varies according to the cultivar, and, therefore, depends mainly on genetic aspects, and ranges from 25 to 35% [29]. However, cultivation conditions such as climatic, geographical and agronomic aspects can influence this percentage even if with a minor contribution [82]. The great interest in hemp fats derives from their high content of unsaturated fatty acids, which reaches almost 90% of the lipid fraction [83]. Their fraction of polyunsaturated fatty acids can reach up to 80% of the total fat [83].

As for many other vegetable oils, hemp seed oil has a high oleic acid content (OA; C18:1; w9) reaching almost 20% [84]. One exception is the Finola variety grown in Italy, which has an OA content that does not reach 10% [55]. OA in a omega 9 monounsaturated fatty acid commonly found in extra virgin olive oil, which is widely used in the Mediterranean area, shows well-known health benefits [85].

Linoleic acid (LA; C18:2; n6) is the main component of hemp seed oil and can exceed 50% of the fat fraction. The intake of LA has a favorable effect on cardiovascular health [86]; however, an excess in LA can limit the pathway of omega 3 biosynthesis through an enzymatic competition process [87,88]. The second most abundant fraction is represented by alpha-linolenic acid (ALA; C18:3; n3). It is the omega-3 fatty acid most represented in plant foods. Among the industrial hemp cultivars, Finola seems to show the highest concentrations of ALA, with percentages reaching 22% [8,55,82,83]. Considering that in a vegetarian diet, the intake of long-chain polyunsaturated fatty acids is marginal, the World Health Organization advises to ensure adequate amounts of ALA to reach the daily recommendations of 1–2% of the total energy intake with omega 3 polyunsaturated fatty acids [89].

Of course, the extraction process must not cause the degradation of the highly reactive double bonds of the carbon scaffold of fatty acids, and for this reason several extraction techniques have been developed, which include the commonly used solvent-extraction technique and mechanical cold-pressing extraction [90].

It is well known that the fractions of omega-6 polyunsaturated fatty acids prevail among polyunsaturated fatty acids in commercially available vegetable oils [35]. At the same time, the main use of vegetable fat sources in the vegetarian diet can only guarantee the supply of short-chain essential fatty acids such as linoleic acid and alpha-linolenic acid, with negligible intakes of long-chain essential fatty acids such as eicosapentaenoic acid, docosahexaenoic acid and arachidonic acid [91,92]. The pool of enzymes capable of converting short-chain fatty acids to long-chain fatty acids is shared by the metabolic pathways of both the 3-series and 6-series of essential fatty acids. This means that in a vegetarian diet, a prevalence of linoleic acid can sequester the enzyme pool by interfering with the elongation and desaturation of alpha-linolenic acid into EPA and DHA. In this respect, it is beneficial in a vegetarian diet to guarantee sufficient sources of omega 3 fatty acids to ensure a proper balance with omega 6 [93,94]. In a vegetarian diet, hemp seed oils can be useful to promote this balance thanks to their omega-3 content. Given the high content in essential fatty acids, two to four tablespoons of hemp seed oil can meet the daily requirement of essential fatty acids in a 2000-calorie diet, providing up to 25 g of LA and 9 g of ALA [95,96].

The main essential fatty acids in hemp are linoleic acid and alpha-linolenic acid, with a beneficial omega 6/omega 3 ratio of 2.5–5.5 [29]. The adequate-intake ratio has been estimated to be 3:1 to 5:1, which reflects the observed proportion in the traditional Mediterranean diet [8,97,98,99]. This ratio is decisive if we take into consideration that in the Western diet, there is a tendency for higher ratios, which is associated with inflammatory events, cardiovascular pathologies and cancer [100,101]. Unfortunately, it has been estimated that this ratio is about 10:1 in the Western diet, and more generally in industrialized countries, due to the prevalence of polyunsaturated fatty acids of the 6 series [102].

A high ratio of unsaturated to saturated fatty acids is also considered protective against cardiovascular disease [103]. The consumption of hemp seed oil can be beneficial to health due to its low content of saturated fatty acids (from 9.4 to 11.7%) [55] and its high content of unsaturated fat, which makes it suitable to adopt an intake of fats with a profitable high PUFA/SFA ratio [104].

**Figure 2:**
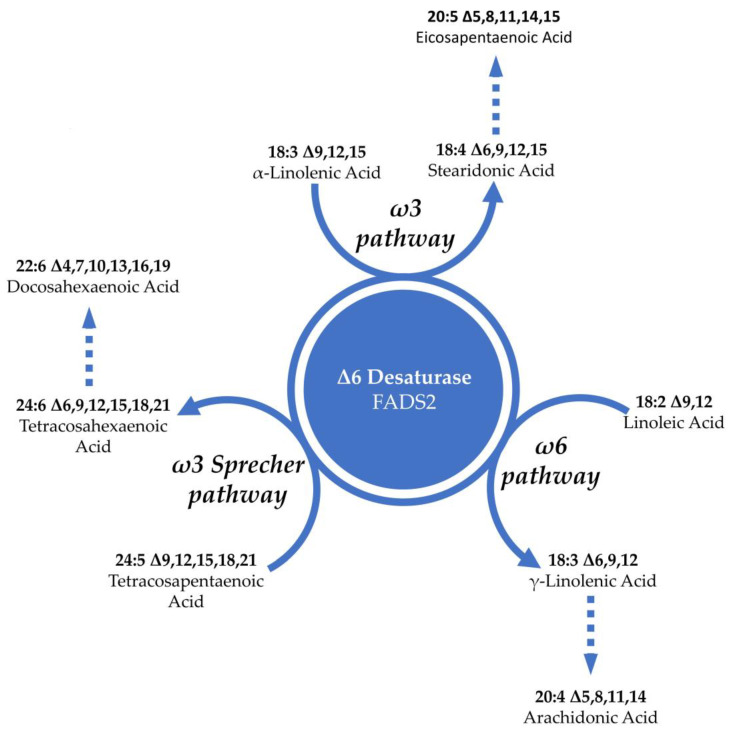
The role of delta-6 desaturase in polyunsaturated fatty acid metabolism.

Another relevant polyunsaturated fatty acid molecule present in hemp is gamma-linolenic acid, known for its anti-inflammatory properties [105]. This fatty acid quickly converts to dihomo-gamma-linolenic acid (DGLA: 20:3; n6). Although hemp seed oil is a good source of GLA, there are other plant sources with higher concentrations such as borage oil, which contains up to 23%, albeit with negligible amounts of omega 3 [106]. In addition to this, hemp, like the entire cannabaceae family, represents a source of stearidonic acid [107]. It is an essential fatty acid of the omega 3 series that is obtained from alpha-linoleic acid through the catalytic action of the enzyme delta-6 desaturase. This enzyme operates on the first step of the polyunsaturated fatty acid maturation pathway and appears to be one of the most rate-limiting steps of the PUFA metabolic pathway [93]. A high intake of linoleic acid could sequester this enzyme by diverting it from the maturation pathway of omega 3 essential fatty acids to the omega 6 series. Furthermore, delta-6 desaturase also participates in the subsequent conversion step of docosapentaenoic acid to docosahexaenoic acid following the Sprecher pathway [108]. An intake of stearidonic acid could bypass this limiting step and favor the biosynthesis pathway of long-chain omega-3 fatty acids [109]. Figure 2 shows the metabolic role of the delta-6 desaturase enzyme in the polyunsaturated fatty acid pathway.

Studies suggest that the fatty acid content varies according to the cultivar. The Finola cultivar shows the most efficient genotype for the formation of gamma-linolenic and alpha-linolenic acid, with the lowest content of saturated fatty acids such as palmitic and stearic, but also with low concentration of oleic acid [82]. As in the case of the ALA and GLA content, the STA content is also higher in Finola compared to other cultivars [8,82,83,102]. This may be particularly relevant in a vegetarian diet where plant sources of omega 3 fatty acids are scant. There are also other plant foods rich in omega 3, mainly seeds and nuts, which can promote a good nutritional balance of omega 3 with omega 6 fatty acids. However, given their limited availability (mainly flax seeds, walnuts and chia seeds), in a vegetarian diet hemp seed oil can also help maintain dietary variability and reach the quota of omega 3 both through the contribution of its alpha-linoleic acid content and also through stearidonic acid intake. It should not be underestimated that hemp seed oil has a particularly high content of essential fatty acids compared to other vegetable oils, and this can shift the preference towards other edible oils, which can contain a higher content of saturated fats that negatively affect human health [8,110]. Even if the presence of polyunsaturated fatty acids favors the oxidation of the hemp oil, the presence in hemp of carotenoids, chlorophyll, sterols and other compounds in the unsaponifiable fraction increases the stability of the extracted oil [111]. Gamma tocopherols, in particular, give a high oxidation stability to hemp seed extracts [29]. However, it has been hypothesized that the antioxidant properties of hemp seeds are related to phenolic compounds in general such as phenylpropionamides (lignans), and more than tocopherols specifically [82]. However, hemp oil degrades easily above 130 °C, and this tendency towards oxidation suggests its use as a condiment oil and not for cooking [30]. However, it shows greater heat resistance than flaxseed oil [112].

While there are numerous preclinical studies on the use of hemp and its derivatives, clinical studies on humans are currently very limited and mostly use hemp seed oil with beneficial effects on atopic dermatitis [113] and multiple sclerosis [114,115,116]. A central role in the health-promoting effect of hemp could be linked to its high content of essential fats and their anti-inflammatory role in the production of eicosanoids (including ceramides) such as SDA, ALA and GLA.

In some clinical trials, hemp seed oil has shown beneficial effects in mental and neurological disorders [117], with a potentially favorable effect on cardiovascular risk factors [118,119].

### 3.3. Dietary Fiber

Dietary fiber can be found the carbohydrate matrix of the seed. Most of the hemp seed fiber resides in the hull and it can be found in both the insoluble and soluble fractions in a ratio of 4:1 [8]. Hemp appears to be one of the most concentrated dietary sources of insoluble fiber [120]. However, industrial transformation processes that expose seeds to high pressures and temperatures (such as extrusion) tend to destroy the structure of the polysaccharides by increasing the proportion of soluble fiber compared to insoluble fiber [121]. The dietary fiber component can range from 27 to 34% [8,122].

The beneficial effects of fiber on human health are well known [123,124,125,126]. Its action is mediated at least in part by the production of short-chain fatty acids (SCFAs) in the bowel lumen by the microbiota, which exhibit anti-inflammatory and immunomodulatory effects capable of contributing to both intestinal and systemic health [127,128,129]. Hemp seeds can help reach the dietary fiber quota recommended, taking into account that in Western countries, there is a reduced consumption of fiber in favor of processed and calorically dense foods [130,131]. However, in a well-planned vegetarian diet, fiber intake is usually sufficient. On the other hand, the excess of fiber, especially in childhood, could interfere with the absorption of other nutrients [132,133,134]. The hulling of hemp seeds can reduce the fiber component and, thus, increase the proportion of other nutrients such as protein and fat fractions. This suggests that, based on an individual’s nutritional needs, the use of hulled or whole seeds should be favored. In a vegetarian diet, hulled seeds could allow for better protein bioavailability due to the increase in digestibility, deriving from the removal of fiber and anti-nutrient compounds present in the hull, limiting the intake of dietary fiber in the overall diet.

### 3.4. Minerals

Hemp seeds are considered a good source of minerals [29]. In a vegetarian diet, some micronutrients can be critical if the diet is not well planned [135,136]. Some critical minerals such as iron, zinc and calcium have been identified. Interestingly, elevated calcium and iron concentrations of up to 955 mg/100 g and 240 mg/100 g, respectively, were detected in hemp seeds [137]. Hemp may, thus, be useful to ensure sufficiently rich sources of calcium to reach the daily requirement of a vegetarian diet [138,139]. Seeds and nuts, legumes, and dehydrated fruit can be good sources of calcium [140]. Among the sources with the best bioavailability of calcium (defined by fractional absorption), we can find cruciferous vegetables such as kale, broccoli, cabbage, cauliflower, mustard and arugula [141]. However, it is unlikely that these foods are represented daily in the diet. Even if the fractional absorption of hemp seeds is not known, consuming different sources of calcium throughout the day can promote enough dietary variability to ensure adequate intake. Despite the above-stated concentration of calcium found in hemp, there is still a wide variability among cultivars, with lower values such as 90 mg/100 g [142].

As far as iron is concerned, it is well known that inorganic iron is less bioavailable than its organic form (heme iron), the latter present in foods of animal origin [143]. It is therefore useful in a vegetarian diet to increase one’s iron intake by up to an additional 80% of the requirement of the general population to compensate for the lower bioavailability [144]. However, in a healthy diet, vegetable sources should be sufficiently represented, limiting the intake of red meat due to health-related risks [145,146,147]. Some guidelines account for low iron bioavailability by implying that a prevalence of plant food must be eaten. In fact, in the fourth revision of the DRV of Nutrients and Energy for the Italian population (LARN) by the Italian Society of Human Nutrition (SINU), as an example of a Mediterranean country, the main source of iron in the Italian diet came from grains [148]. Heme iron is more prone to create oxidative stress through the formation of free radicals through Fenton reactions [149,150,151]. Along with other iron-rich plant foods, such as legumes and whole grains, hemp can help meet the daily requirement. In the literature, the iron concentrations detected for hemp seeds are highly variable [8,102]. Values up to 240 mg/100 g can be justified by iron-rich soils used experimentally for the growth of hemp [137]. Nevertheless, the iron content in hemp seeds is higher than that of any other grains [84]. Unlike grains, whose iron concentration seems higher in the case of whole grain foods, the hulling of hemp seeds increases the net percentage of iron found (up to 25% more) and allows the zinc content to double [142], even if not all researchers confirm this hypothesis [122].

Overall, using hemp seed flour can be an excellent source of minerals with wider applications.

### 3.5. Bioactive Molecules

Hemp is rich in phenolic compounds but the levels may vary according to the cultivar [30]. The anatomical part of the seed containing the greatest amount appears to be the hull, with smaller concentrations in the kernel [152]. However, a greater radical-scavenging capacity was found in the cotyledon fraction compared to the hull. It seems that hemp pectin can create a matrix that traps polyphenols in a continuum dispersed-liquid phase [153]. Hydrogen bonds and hydrophobic bonds explain the interactions between dietary fiber and water-soluble phytochemicals [154]. Based on these observations, hemp oil could be the hemp derivative with the lowest total phenolic content, with meal showing intermediate concentrations compared to the hull and oil [155]. The exposure of the sprouts to specific radiations can increase the content of flavonoids and polyphenols, and, therefore, improve their antioxidant activity compared to raw seeds [156].

The two classes of compounds that have attracted the most interest for potential antioxidant effects are hydroxycinnamic acids and lignanamides. The latter are classified into Cannabisins and other compounds based on the type of lateral residues present in the molecular structure, and include about 20 different molecules [30].

The unsaponifiable fraction of hemp represents less than 2% of the oil and includes sterols, tocopherols and water-soluble vitamins, which show beneficial effects for human health and could lead to the greater consumption of hemp for health purposes [157]. For example, hemp sterols, mainly beta-sitosterol, represent about 15% of the unsaponifiable fraction of hemp oil; in comparison, olive oil contains about half of this concentration [157].

Phytosterols have a structure similar to cholesterol but cannot be synthesized by humans and can only be found in plants. Their beneficial feature for health is to modify the solubility of cholesterol in the intestine, reducing its absorption through a process of exclusion from the lipid fraction, through a mechanism of competition in the lipid micelles [158]. Although the literature on the subject is scarce, the phytosterol component has been quantified in almost 280 mg/100 g in the oil [157] and about 124 mg in the whole seed [159]. The most representative isoform in hemp is beta-sitosterol, which ranges from 190 to 54 mg/100 g with the highest values in the case of the oil compared to the seed [102,157,159]. In addition to its cholesterol-lowering effect, anti-inflammatory and antineoplastic effects have been described [159]. Along with pistachio, hemp appears to be one of the richest sources of beta-sitosterol [29].

Among the carotenoids present in hemp, lutein is the most abundant (1.4–3.4 mg/100 g of the whole hemp seed) [82]. Like zeaxanthin, lutein accumulates in macular cells, showing protection against light-induced oxidative stress [160,161].

Furthermore, hemp oil contains about 80 mg/100 g of total tocopherols: plant-derived fat-soluble molecules with antioxidant activity that can be found in foods. Hemp seed oil is one of the most concentrated sources of these phytochemicals [162]. Thanks to their free-radical scavenger action, they play a crucial role in preserving hemp oil from oxidative stress, and can be found in different isomers such as the most represented delta-tocopherol. The concentrations of this isomer in hemp seed oil are higher than in sesame and sunflower oil [142]. It seems that this isomer has the best antioxidant activity in lipid matrices compared to others [103]. The literature is very heterogeneous regarding the concentrations of tocopherols in hemp, with amounts ranging from 14 to 135 mg/100 g depending on the matrix studied (oil or the whole seed) or on the extraction method [29]. Similarly, the presence of delta-tocopherol ranges from 0.5 to 116 mg/100 g. Instead, alpha-tocopherol is less represented in hemp. Despite the heterogeneous concentrations of phenolic compounds in hemp seed oil, its antioxidant power seems to be higher than flaxseed oil, soybean oil, and grapeseed oil [163].

Other molecules such as sativamides may have potential use in neurodegenerative diseases such as Parkinson’s and Alzheimer’s disease [164]. Furthermore, some phenolic compounds extracted from hemp have shown arginase inhibition activity, with a consequent increase in nitric oxide levels, which can improve endothelial function in cardiovascular diseases [165].

Currently, the HEMPEDOCLE observational study organized by a REICA and sponsored by Istituto Zooprofilattico Sperimentale del Mezzogiorno (Italy) is aiming to evaluate the health effects of the use of cannabis and its derivatives on hundreds of users, which includes the use of hemp-based foods, supplements and nutraceuticals [166].

Table 3 summarizes the main nutritional characteristics of hemp.

## 4. Sustainability

The environmental impact of foods is an increasingly debated topic due to the awareness of the media and the scientific literature. It is well known that human activities have an impact on the planet through the exploitation of resources that do not renew sufficiently to guarantee an adequate turnover and, at the same time, through the release of compounds that favor the greenhouse effect and pollute land and water. Among the various activities, in recent decades it has emerged that food production participates towards the environmental impact, and the exploitation of food resources show a non-negligible impact, if analyzed along their entire supply chains [167,168,169]. The vegetarian choice is driven by many factors such as health and ethics, but the ecological and environmental sustainability aspect is part of a holistic concept that also includes human health and social sustainability [16,170]. The use of hemp for food purposes can be consistent with the vegetarian diet both because it can represent a plant-based source of nutrients useful for supporting nutritional needs and health, and for the possible advantageous implications of its cultivation on the environmental impact of agriculture. Compared to other crops, hemp appears to have a lower environmental impact, with the ability to grow rapidly and efficiently sequester carbon [171,172,173]. The rapid growth limits the need for the use of herbicides, fungicides and other pesticides used for pest and weed control [172,173,174]. Furthermore, its rapid exfoliation allows the efficient recovery of the cultivated soils [171,175] and makes hemp an ideal plant for crop rotations in order to avoid overexploitation of the soil [173]. Furthermore, hemp shows reduced requirements regarding the need for irrigation [172].

The use of hemp as a phytoremediation technique has been described, especially for the removal of heavy metals [137]. However, the translocation of heavy metals such as cadmium to the seed raises concerns about the use of hemp for soil decontamination and concomitant food use. This phenomenon is amplified in the case of the use of fertilizers [137].

## 5. Hemp Derivatives

Hemp shows a wide versatility of use, especially by derivatives used for nutritional and functional applications.

In Canada, there are several companies such as Fresh Hemp Foods, Natures Path, Hemp Oil Canada and Ruths Hemp Foods, which have developed hemp-derived foods such as edible oils, snacks and flour [176].

Macronutrients including dietary fiber and protein can be included in bread products to replace other sources of nutrients or to confer additional characteristics such as a reduction in cooking times and volume losses of bakery products [177,178,179]. Hemp protein isolates at a concentration of 30 g/L have been added to cranberry juice to increase the concentration of antioxidants through proanthocyanidin stabilization [180], or to obtain infant formula [181]. Hemp can be used as a fortifying and enriching agent for rheological and technological purposes in the bakery industry. For example, the addition of an extruded product from 5% to 15% *w*/*w* (including 30% hemp seeds and 70% rice flour) or hemp seed flour from 10% to 20%, were employed to increase the volume of a bread dough [182,183]. Other properties of doughs containing hemp affect the curb and crust bread color of the fortified bakery products if present at 15% in the form of extruded or non-extruded hemp-rice flour [184]. Hemp can also be added as hemp seed flour or whole hemp seed powder at 20% *w*/*w* to the dough to improve its nutritional characteristics by increasing antioxidants, calcium, iron, protein, fiber and fat, as it also can be in other processed foods [177,182,185]. Its use in the bakery sector is particularly interesting in the case of the formulation of products for celiac people, being naturally gluten-free, as it improves the acceptability and nutritional value of gluten-free products by replacing gluten-free starch with hemp flour or proteins at a ratio ranging from 10% to 60% [179,186].

As discussed above, the presence of essential fatty acids in hemp is a major nutritional-profile aspect. The extraction of the oil from the seeds can take place using different methods, which lead to variations in the yield and composition of the final product. Although mechanical cold pressing is the most commonly adopted, ultrasonication and supercritical fluid extraction show the best yields from a quantitative and economic point of view, respectively. Instead, to obtain the best composition of polyunsaturated fatty acids, it is necessary to apply the Soxhlet extraction [90].

The high concentration of polyunsaturated fatty acids makes hemp oil prone to oxidation. However, pretreatment with hydrolytic enzymes can improve the stability of the oil subsequently obtained due to the release of antioxidant phytochemicals from the cell structures [187]. The extraction of oil using ultrasound can have a positive influence on the quality of the oil obtained, limiting the oxidative damage through the inhibition of radical reactions [188].

Based on the organization of the production and transformation chain, it must be taken into consideration that some oil-extraction processes, such as solvent extraction, could profoundly alter the amino acid composition of the hemp seed cake, influencing the possibility of reusing this transformation byproduct to obtain a nutritional source, especially as a protein source [189]. Conversely, extraction with alkali does not involve amino acid deterioration of the hemp protein fraction [190]. Replacing 20% and 50% of wheat flour with hemp seed flour can increase the protein content by 38% and the polyphenol content by 400%, respectively, of bread products [177,185].

Three protein-rich products may be derived from hemp:

- Hemp protein meal (HPM): obtained by crushing hemp seeds after oil extraction.
- Hemp protein concentrate (HPC): obtained from hulled and defatted seeds. It can be obtained from HPM by further processing.
- Hemp protein isolate (HPI): this is the most purified form through extraction techniques that provide chemical–physical processing, aimed at optimizing the precipitation of the proteins and removing the non-protein fractions.

The protein content in said hemp products also varies according to the cultivar used, growing conditions and processing techniques. HPM contains from 30 to 50% of protein of the dry weight [32]. In HPC, the protein content reaches 65% of the dry weight and can reach 70% in the case of the use of an enzymatic pre-digestion process on the fiber and subsequent ultrafiltration [49]. Protein enrichment also leads to an improvement in protein bioavailability by increasing digestibility. The hemp seed product with the highest protein concentration is HPI, which can even exceed 90%. It can be used to obtain protein-enriched products with additional functional properties. Among the most commonly used extraction systems to obtain HPI is extraction with alkali and subsequent precipitation [47,65,74,191]. Other extraction methods are acid extraction, salt extraction and micellization. The latter can reach high levels of purity up to 99% of protein on dry weight [192]. The degree of protein purity of hemp products affects the protein quality itself. Thus, HPC has a higher concentration of branched-chain amino acids and, more generally, essential amino acids than HPM [193]. This can be of interest in the case of a vegetarian diet, especially to support an intense physical effort by vegetarian athletes. 

Even if they are not widespread yet, hemp-based meat analogues can be obtained, as has already been widely developed with other vegetable sources (soy, pea, wheat proteins), mycoproteins and other texturized vegetable proteins [194]. Hemp proteins can also be used in conjunction with other proteins, usually used for meat analogues such as soy, to improve the characteristics of the extrusion products by substituting soy protein isolate with hemp protein concentrate in a mixture up to 60% *w*/*w* [194]. Furthermore, hemp can be used to obtain plant-based milk rich in proteins and essential fats and low in carbohydrates [195]. Through specific pasteurization processes, it was possible to obtain hemp milk while avoiding the development of bitterness or dark color that could negatively influence consumer acceptability [181]. The frontier of plant-based milk is very promising, and consumers are showing a strong interest in vegetable alternatives to cow’s milk that has stimulated a re-evaluation of the nomenclature of these products, towards which regulatory bodies such as the FDA have showed a conservative approach in the past [196,197]. Hemp seeds have been used for various food preparations such as gnocchi, biscuits, chips, crackers, cookies, pasta, and energy bars, including gluten-free versions as discussed above [6]. A recent pilot study showed an improvement in pain relief in patients with osteoarthritis after arthroplasty surgery following the consumption of pasta enriched with 15% hemp seed flour [198]. The effect of hemp seeds on bone tissue was confirmed by in vitro evaluation of bone metabolism. The daily consumption of a 2 g hemp seed-powder food supplement in combination with aerobic activity for 8 weeks showed improvement in parameters related to oxidative stress (CAT), BDNF and lipid profile (HDL) in sedentary young men [199]. Hemp seeds were also used to formulate a seasoning source obtained using fermentation [200]. Collectively, the hemp and hemp-based food market is projected to grow to USD 11.59 billion in 2029 [201]. This projection is in agreement with recent works that showed a positive propensity of consumers towards the use of hemp-based products [202]. The increased projections seem to be driven by the interest in new foods and also by the adoption of vegetarian and vegan diets [203]. Recently, CANNUSE, an official database to collect information about the use of cannabis, was created [204]. Among the 31 countries surveyed around the world, India and Pakistan seem to be the major users of cannabis through their traditional medicine, and CANNUSE lists over 2000 items for food use, representing 7.3% of total items. Seeds account for 15% among the anatomical part of the plant used for any purpose. A total of 40% of seeds are used for traditional food and drinks [205].

Table 4 highlights the discussed application for hemp products and derivatives.

## 6. Regulatory Aspects

Biotypes or chemotypes allow *C. sativa* varieties to be classified according to their cannabinoid content, which is also the aspect of greatest concern [206,207,208]. Classifications distinguish biotypes with a high THC content and low CBD content; a biotype with a high CBD content and low THC content; and, finally, an intermediate biotype between the previous two, showing a homogeneous ratio between THC and CBD. The first biotype is used for narcotic purposes and is therefore defined as the “drug type”. The second biotype has an industrial use (or fiber-type), while the intermediate biotype is mainly used for medical purposes [209]. THC has a toxic effect and is used as a narcotic, whereas CBD is a cannabinoid mainly used in medicine without any major psychoactive effects. THC and CBD are the two major phytochemicals present in *C. sativa*, although at least 100 cannabinoid phenolic compounds typical of this plant have been found [210]. While in the early 20th century, hemp was widely cultivated for textile purposes [13], and Italy and Russia were the major European producers [103], later concerns about the effects of THC fostered the ban of all types of hemp without distinctions of uses for textile applications from narcotic use. Similarly, directives were issued in the European Union to limit psychotropic substances such as THC [211]. Only in 1994 did Canada, among the first countries to reintroduce it and currently among the major producers, authorize the cultivation of *C. sativa* with THC concentrations lower than 0.3%. In Europe, only more recently (in 2013), EU regulation 1207 was issued which allows the cultivation of *C. sativa* for industrial purposes based on a THC content of less than 0.3% in flowers and leaves [212]. An extensive list of over 50 permitted varieties has been listed for cultivation use in the European Plant Variety Database [213]. Today, many European countries show an intense production of hemp, so Europe ranks second in terms of production after Canada [13]. In 2018, the US hemp cultivation policy was also changed with a new federal regulatory system in which hemp cultivars with THC concentrations of less than 0.3% were removed from DEA prohibition and supervision to facilitate trading [13].

Cannabinoids such as tetrahydrocannabinol (THC), cannabidiol (CBD) and cannabinol (CBN) are obtained indirectly from cannabigerolic acid (CBGA) through the heat decarboxylation of its metabolites cannabidiolic acid (CBDA) and tetrahydrocannabinolic acid [214]. CBGA is a secondary metabolite of *C. sativa* derived from the inflorescences of the plant. This means that the seeds do not contain compounds that can be converted into psychoactive substances. Traces of THC can be found in the seeds due to contamination by the resins of the plant. Even if the discussion of psychoactive substances is beyond the scope of this review, we must not forget that the presence of THC affects the regulations of the states and, therefore, the farming and marketing of hemp and hemp products also for food use. Furthermore, there is still great confusion in public opinion about the difference between industrial hemp, normally used for textile and food purposes, and marijuana. This has created a bad reputation, which limits the diffusion of a plant with interesting properties both for health and nutrition.

THC is a substance of particular interest in the recreational use of hemp. Concentrations below 1% have been found in hemp seeds, and its presence is caused by inadequate cleaning of the seeds that come into contact with the epidermal glands that secrete the resins [215]. The presence of various cannabinoids can also vary according to the chemical–physical conditions of the transformation processes, such as the extraction conditions that employ high temperatures and favor the auto-decarboxylation of the hemp metabolites [30]. Numerous cannabinoids have been identified in hemp oil [216].

## 7. Future Perspectives and Research Directions

Compared to soy protein isolate, hemp protein has a low solubility, which complicates the use of this protein source in some applications [64]. This characteristic could depend on the aggregation capacity of the high number of disulphide bonds along the molecular scaffold of the protein. The selection of the manufacturing processes aimed to solve this type of limitation could increase the use of hemp in the food sector.

There are novel techniques for extracting oil from hemp seeds which, as we have seen, could improve the yield and quality of the products obtained. Although mechanical cold-pressed extraction is the most widespread and simple to adopt, other modern techniques are very promising. However, they require technological development and know-how adaptation by the food industry. Furthermore, it should be considered that in Europe, the marketing of food products obtained with innovative techniques would fall under the regulation of novel foods and, therefore, a prior safety assessment is needed [217]. Hemp seeds, oil and hemp protein are currently considered Generally Recognized as Safe (GRAS) by the U.S. Food and Drug Administration and may be used as an ingredient or food additive in food for human consumption without further permit requirements [218].

The interest in hemp polyphenols and their antioxidant properties represents a starting point for the application of transformation techniques that favor the detachment of phytochemicals from the matrix of hemp fiber which, through chemical interactions of various kinds, limit their intestinal bioavailability. The extraction of polyphenols from the seeds along the transformation process could increase the value of the hemp-based products and its interest in human health applications. Micro- and nano-encapsulation could protect the polyunsaturated fatty acids contained in hemp seed oil from oxidation [219,220]. This encapsulation could also be useful for conveying essential amino acids such as arginine, which could be recovered from other processing chains such as that of textile hemp fibers [193,221]. However, attention must be paid to obtaining processes that are food-grade and that, at the same time, can supply a recovery strategy of byproducts from other supply chains already widely exploited industrially.

The new frontier of bioactive peptides suggests that hemp could be a source of substances useful for the treatment of metabolic pathologies, thanks to its antioxidant and ace-inhibitory effects that could improve cardiovascular pathologies and hypertension. However, it should be clarified whether these beneficial effects can be partly driven by the consumption of hemp seeds and their derivatives as food. Although the literature is rich in information about the nutritional aspects of hemp seeds and their derivatives, data on health effects remain fragmented. The standardization of the contents of the bioactive compounds found in hemp seeds and their derivatives, as well as the concentration sufficient to obtain the alleged benefits, is a gap in the literature that must be filled to stimulate the use of hemp for nutraceutical use.

**Figure 3:**
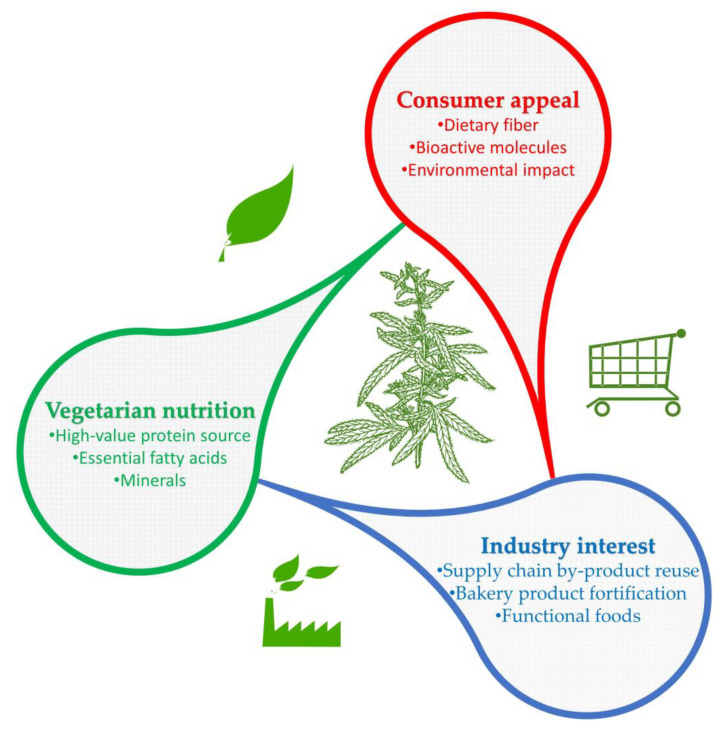
Graphical representation of promising hemp use.

Finally, even if hemp has a long history of human use sufficient to consider it safe, some concentrated extracts should pass the verification of safety for use in humans, which has still not been fully clarified. Figure 3 shows possible applications of hemp that deserve greater development.

## Figures and Tables

**Table 1:** Selected hemp features and related benefits.

Hemp Feature	Benefits
Protein	The high nutritional value of hemp protein can help one to reach the adequate intake of protein in a vegetarian diet without animal sources.
Essential fatty Acids	A vegetarian diet has limited sources of EFAs, especially omega 3. The advantageous ratio of n6/n3 in hemp oil can help to obtain a balanced intake of polyunsaturated fatty acids.
Calcium	There are limited sources of calcium in a vegetarian diet without milk and dairy usage. Using various plant foods including hemp can help to reach the RDA for calcium.
Iron	In a vegetarian diet, there are only low-bioavailable sources of iron so different iron-rich foods must be used, and hemp can have a high content of this mineral.
Fiber	Even if all plant-based foods are rich in dietary fibers, the functional properties of insoluble fiber from hemp can stimulate its consumption.
Phytochemicals	The high content of bioactive molecules with health effects can stimulate the consumption of hemp in a plant-based context.
Environmental impact	Considering the wide motivation for a vegetarian diet adoption, the low impact of hemp cultivation can prompt the use of hemp as an eco-friendly plant-based source.
Versatility	Hemp can have promising features, and the seeds can be employed in various industrial productions including some products of interest for a vegetarian diet such as plant-based milk and meat alternatives. Moreover, employed as a fortifier, hemp derivatives can be used in supplement and bakery products.

**Table 2:** Nutritional composition of selected foods *.

Food	Energy (Kcal)	Protein (g)	Arg (g)	Fat (g)	SFA (g)	MUFAs (g)	OA (g)	PUFAs (g)	LA (g)	ALA (g)	SDA (g)	Ca (mg)	Fe (mg)
Hemp seeds	553	31.56	4.55	48.75	4.6	5.4	5.276	38.1	27.459	10.024	0.617	70	7.95
Adzuki beans	329	19.87	1.284	0.53	0.191	0.05	0.05	0.113	0.113	NA	NA	66	4.98
Almonds	579	21.15	2.465	49.93	3.802	31.551	31.294	12.329	12.324	0.003	0	269	3.71
Brazil nuts	659	14.32	2.14	67.1	16.134	23.879	23.594	24.399	24.363	0.036	0	160	2.43
Cashew nuts	553	18.22	2.123	43.85	7.783	23.797	23.523	7.845	7.782	0.062	0	37	6.68
Chestnuts	213	2.42	0.173	2.26	0.425	0.78	0.749	0.894	0.798	0.095	NA	27	1.01
Chia seeds	486	16.54	2.143	30.74	3.33	2.309	2.203	23.665	5.835	17.83	NA	631	7.72
Chickpeas	378	20.47	1.939	6.04	0.603	1.377	1.365	2.731	2.629	0.102	0	57	4.31
Fava beans	88	7.92	NA	0.73	0.118	0.104	0.097	0.342	0.312	0.03	NA	37	1.55
Flaxseed	534	18.29	1.925	42.16	3.663	7.527	7.359	28.73	5.903	22.813	0	255	5.73
Kidney beans	333	23.58	1.46	0.83	0.12	0.064	0.064	0.457	0.178	0.279	0	143	8.2
Lentils	352	24.63	1.903	1.06	0.154	0.193	0.184	0.526	0.414	0.112	0	35	6.51
Lima beans	338	21.46	1.315	0.69	0.161	0.062	0.052	0.309	0.215	0.095	0	81	7.51
Lupins	371	36.17	3.877	9.74	1.156	3.94	3.558	2.439	1.995	0.446	NA	176	4.36
macadamia nuts	718	7.91	1.402	75.77	12.061	58.877	43.755	1.502	1.296	0.206	0	85	3.69
Mungo beans	341	25.21	1.642	1.64	0.114	0.085	0.085	1.071	0.072	0.999	NA	138	7.57
Navy beans	337	22.33	1.02	1.5	0.17	0.128	0.117	0.873	0.335	0.538	NA	147	5.49
Peanuts	567	25.8	3.085	49.24	6.279	24.426	23.756	15.558	15.555	0.003	0	92	4.58
pecan nuts	691	9.17	1.177	71.97	6.18	40.801	40.594	21.614	20.628	0.986	0	70	2.53
Pine nuts	673	13.69	2.413	68.37	4.899	18.764	17.947	34.071	33.15	0.164	0	16	5.53
Pistachio	560	20.16	2.134	45.32	5.907	23.257	22.674	14.38	14.091	0.289	0	105	3.92
Soybeans	446	36.49	3.153	19.94	2.884	4.404	4.348	11.255	9.925	1.33	NA	277	15.7
Walnuts	654	15.23	2.278	65.21	6.126	8.933	8.799	47.174	38.093	9.08	0	98	2.91

* NA: Not Available.

**Table 3:** Nutritional aspect of hemp seed.

Nutrient	Fraction	Whole	Dehulled	Meal	Hull	Oil
Protein	Total (%)	21–28	36	41	13	
	Arginine (%)	2.28–3.10	4.55	3.91	0.94	
Lipids	Total (%)	24–36	47	10	10	100
	PUFA (%) LA (%) ALA (%) SDA (%) n6:n3					72–84 52–59 10–22 0.2–2 2.5–5.5
Fiber	Total (%)	28–34	8	30	65	
	Insoluble (%)	22–31				
	Soluble (%)	3–5				
Sterols	Total (mg/100 g) β-Sitosterol	124 54–80				279 190
Tocopherols	Total (mg/100 g) γ-Tocopherol (mg/100 g)	61–135 1–295				14–97 15–89
Minerals	Ca (mg/100 g)	90–955				
	Fe (mg/100 g)	4–240				

**Table 4:** Application of hemp products.

Hemp Derivative	Application	Main Features
Protein isolate	Juice fortification, infant formula, plant-based-milk and supplement manufacture	Increasing oxidative stability and protein-rich source
Extruded product or seed flour	Production of bakery products, pasta, energy bars and meat analogues	Increasing dough volume and color Increasing mineral, protein, fat, fiber and antioxidant content Application to gluten-free products
Seeds	As food for salads and soups, or seasoning production	Nutrient source
Seed oil	Table oil (not for cooking)	Polyunsaturated fatty acid and antioxidant nutritional source

## Data Availability

Not applicable.
